# Early outcomes of “low-risk” patients undergoing lung resection assessed by cardiopulmonary exercise testing: Single-institution experience

**DOI:** 10.3389/fsurg.2023.1130919

**Published:** 2023-03-16

**Authors:** Riccardo Orlandi, Rocco Francesco Rinaldo, Alessandra Mazzucco, Andrea Baccelli, Michele Mondoni, Francesca Marchetti, Mariapia Zagaria, Jacopo Cefalo, Andrea Leporati, Matteo Montoli, Giorgio Ghilardi, Alessandro Baisi, Stefano Centanni

**Affiliations:** ^1^ Department of Thoracic Surgery, University of Milan, Milan, Italy; ^2^ Respiratory Unit, ASST Santi Paolo e Carlo, San Paolo Hospital, Department of Health Sciences, University of Milan, Milan, Italy; ^3^ Thoracic Surgery Unit, ASST Santi Paolo e Carlo, San Paolo Hospital, University of Milan, Milan, Italy; ^4^ General Surgery Unit, ASST Santi Paolo e Carlo, San Paolo Hospital, University of Milan, Milan, Italy

**Keywords:** cardiopulmonary exercise testing (CPET), thoracic surgery, postoperative outcomes, VO_2_peak, peak oxygen uptake, cardiopulmonary complications

## Abstract

**Objective:**

Cardiopulmonary exercise testing (CPET) is currently recommended for all patients undergoing lung resection with either respiratory comorbidities or functional limitations. The main parameter evaluated is oxygen consumption at peak (VO_2_peak). Patients with VO_2_peak above 20 ml/kg/min are classified as low risk surgical candidates. The aims of this study were to evaluate postoperative outcomes of low-risk patients, and to compare their outcomes with those of patients without pulmonary impairment at respiratory function testing.

**Methods:**

Retrospective monocentric observational study was designed, evaluating outcomes of patients undergoing lung resection at San Paolo University Hospital, Milan, Italy, between January 2016 and November 2021, preoperatively assessed by CPET, according to 2009 ERS/ESTS guidelines. All low-risk patients undergoing any extent surgical lung resection for pulmonary nodules were enrolled. Postoperative major cardiopulmonary complications or death, occurring within 30 days from surgery, were assessed. A case-control study was nested, matching 1:1 for type of surgery the cohort population with control patients without functional respiratory impairment consecutively undergoing surgery at the same centre in the study period.

**Results:**

A total of 80 patients were enrolled: 40 subjects were preoperatively assessed by CPET and deemed at low risk, whereas 40 subjects represented the control group. Among the first, 4 patients (10%) developed major cardiopulmonary complications, and 1 patient (2.5%) died within 30 days from surgery. In the control group, 2 patients (5%) developed complications and none of the patients (0%) died. The differences in morbidity and mortality rates did not reach statistically significance. Instead, age, weight, BMI, smoking history, COPD incidence, surgical approach, FEV1, Tiffenau, DLCO and length of hospital stay resulted significantly different between the two groups. At a case-by-case analysis, CPET revealed a pathological pattern in each complicated patient, in spite of VO_2_peak above target for safe surgery.

**Conclusions:**

Postoperative outcomes of low-risk patients undergoing lung resections are comparable to those of patients without any pulmonary functional impairment; nonetheless the formers represent a dramatically different category of individuals from the latter and may harbour few patients with worse outcomes. CPET variables overall interpretation may add to the VO_2_peak in identifying higher risk patients, even in this subgroup.

## Introduction

Surgery is the treatment of choice for early-stage Non-Small Cell Lung Cancer (NSCLC) and for selected cases of locally advanced-stage NSCLC, since radical lung resection is nowadays a potentially curative therapy in lung cancer (1). However, surgery carries a significant burden of morbidity and mortality; therefore, patients’ fitness for the intervention should be considered; otherwise, other medical or less-invasive therapies should be offered. In addition, since most lung cancers patients are smoking elderly with several comorbidities, a thorough preoperative evaluation is mandatory to refer each patient to the best possible treatment (2). Both European Respiratory Society (ERS) (3) and American College of Chest Physicians (ACCP) (4) have recommended that each surgical candidate should first undergo cardiac assessment and then pulmonary function testing to estimate perioperative risk. Currently, Cardiopulmonary Exercise Testing (CPET) is recommended by both Societies in patients with respiratory comorbidities and/or functional limitations to further stratify their perioperative risk. CPET can be defined as a holistic physiologic testing, assessing the whole patient’s respiratory, cardiovascular, and metabolic response to stress. ERS (3) suggests that each patient with either forced expiratory volume in 1 s (FEV1) or diffusing lung capacity for carbon monoxide (DLCO) <80% predicted should undergo CPET; ACCP (4) recommends performing CPET in patients with either predicted postoperative(ppo)FEV1 or ppoDLCO <30%, or in case of unsatisfactory performance in a low technology exercise test. Oxygen consumption at peak (VO_2_peak) is acknowledged as the most important variable for risk stratification: for values of VO_2_peak >20 ml/kg/min, <20 ml/kg/min and >10 ml/kg/min, <10 ml/kg/min, risk of complications is considered low, moderate, and high, respectively. The low-risk group could potentially undergo lung resection up to pneumonectomy, without any increased risk; nonetheless, literature is still lacking univocal information on the outcomes and prognostic significance of this specific group. The primary aim of this study was firstly to evaluate postoperative outcomes (mortality and morbidity rates) of patients undergoing surgical lung resection and deemed to be at low risk on preoperative CPET. We also compared their outcomes with those of patients with normal pulmonary function testing, who are by definition standard-risk subjects.

## Materials and methods

### Study design

This is a retrospective, monocentric, observational study, analyzing consecutive patients who were preoperatively assessed through CPET, and underwent surgical lung resection for pulmonary nodules between January 2016 and November 2021, at San Paolo University Hospital in Milan, Italy. A case-control study was nested, comparing outcomes of enrolled patients preoperatively assessed by CPET with those of consecutive patients matched 1:1 for type of surgery, who had undergone surgical lung resections in the same time lapse at the same center without need for CPET. The study was conducted in accordance with the Declaration of Helsinki, and it was reviewed and approved by Milan Area 1 ethics committee.

### Study population

Inclusion criteria were: age ≥18 years, preoperative CPET because of higher surgical risk according to 2009 ERS/ESTS Guidelines algorithm (either FEV1 or DLCO <80% predicted), VO_2_peak from CPET above 20 ml/kg/min, lung resection of any extent for established or suspected lung neoplasia with radical intent.

Exclusion criteria were inoperability and VO_2_peak from CPET below 20 ml/kg/min.

Inclusion criteria for patients of control group were: age ≥18 years, preoperative FEV1 and DLCO ≥80%, lung resection of any extent for established or suspected lung neoplasia with radical intent.

Exclusion criteria for patients of control group were inoperability and need for CPET.

Preoperative, intraoperative, and postoperative data were prospectively collected into institutional database and retrospectively analyzed.

### Pre-admission exams

All patients routinely underwent clinical history questioning, physical examination, blood tests, 12-lead electrocardiogram (ECG), pulmonary function testing (PFT) according to ERS/American Thoracic Society (ATS) (5) guidelines using the Quark PFT modular system (Cosmed, Rome, Italy), arterial blood gas analysis (BGA), CPET.

### Cardiopulmonary exercise testing

Patients underwent symptom limited CPET using bicycle ergometer and breath-by-breath gas exchange analysis was performed through medical system respiratory analyzer (Sensormedics, Vmax Spectra®, Yorba Linda, United States). Incremental protocol was applied: the increasing ramp-pattern rate (Watts/minute, W/min) has been determined individually, based on rest functional data and expected exercise tolerance, to achieve exhaustion between 8 and 12 min. All CPETs were performed at Department of Pneumology of our institution. During CPET the following parameters have been constantly measured: 12-lead electrocardiogram (ECG), gas-exchange by mouthpiece, heart rate, pulse oxygen, arterial blood pressure (6). An arterial blood sample was collected for blood gas analysis (BGA) and lactates at the peak of the exercise, as soon as patients reported to be exhausted, as previously described (7). We defined preserved exercise capacity when VO2peak ≥85% of predicted; we considered an upper limit of normal for the minute ventilation to carbon dioxide production (VE/VCO_2_) slope of 30 (8).

### Surgical procedures

Surgical resections were performed by the same team of three experienced thoracic surgeons: sub-lobar resections (segmentectomies and wedge resections), lobectomies, bi-lobectomies and pneumonectomies were included. Operations were performed though lateral muscle-sparing thoracotomy or bi-portal video-assisted thoracoscopy. Perioperative management followed standardized pathways of care.

### Mortality and morbidity

Mortality is defined as in-hospital death or death within 30 days from surgery. Morbidity is defined as occurrence of major cardiopulmonary complications within 30 days from surgery: bronchopulmonary infections or pneumonia (typical clinical, laboratory and radiographic features), respiratory failure (partial arterial oxygen pressure <60 mmHg and/or partial arterial carbon dioxide pressure >45 mmHg) needing for mechanical ventilation, acute respiratory distress syndrome (based on Berlin criteria), atelectasis requiring bronchoscopy or mechanical ventilation, pulmonary oedema, pulmonary embolism, arrhythmia (hemodynamically unstable and requiring treatment), acute myocardial ischemia, cardiac failure needing for inotropic support, pulmonary sepsis or multi-organ failure (9, 10).

### Statistical analysis

Primary outcomes were postoperative mortality and major cardiopulmonary morbidity, occurring within 30 days from surgery. Normality was confirmed for all the continuous variables, as assessed through the Kolmogorov–Smirnov test and visual plot analysis. Continuous variables are expressed as means and standard deviation, whereas nominal variables are presented as numbers with percentages. Furthermore, exploratory univariable analysis was performed to detect differences between patients deemed at low-risk by CPET and patients without need for CPET: continuous variables with normal distribution were compared using unpaired Student’s t-test, whereas nominal variables were compared using chi-squared or Fisher’s exact test, whenever appropriate. Data were analyzed using Statistical Package for Social Sciences (SPSS Inc., Chicago, United States), version 23.0. Values of *p* < 0.05 were considered statistically significant.

## Results

**Figure 1:**
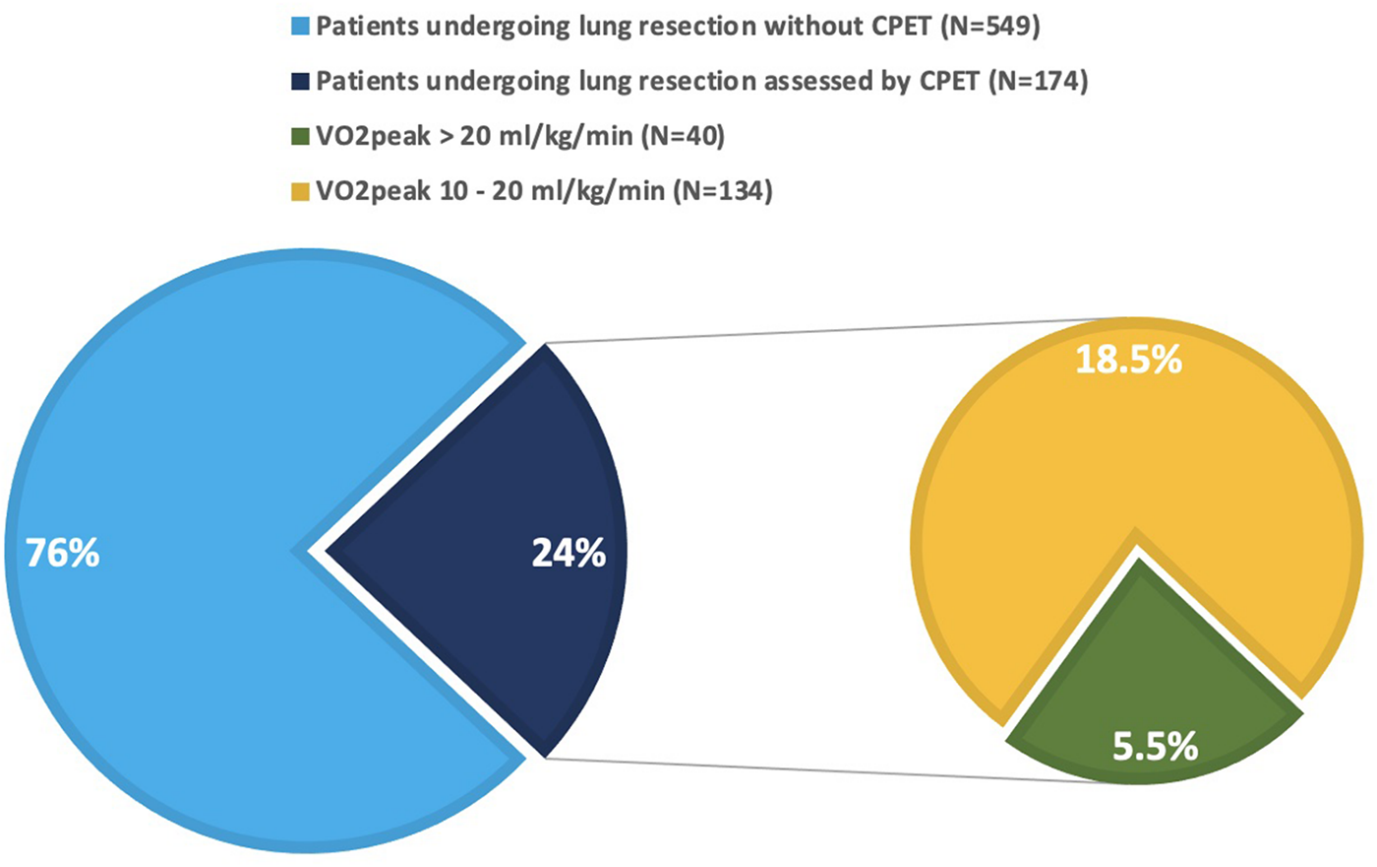
Patients undergoing lung resection for pulmonary nodules during the study period. Green slice represents the patients included in the analysis (5.5% of the whole population, 23% of the patients assessed by CPET).

Globally, 723 patients underwent pulmonary resections for established or suspected lung cancer at the reference hospital during the study period. 174 (24%) were preoperatively assessed by CPET, due to either FEV1 or DLCO <80% predicted, according to 2009 ERS/ESTS guidelines (3), as presented in Figure 1. Among them, 40 patients (23%) had VO_2_peak above 20 ml/kg/min, hence deemed to be at low risk for postoperative complications and were included in the analysis. The cohort population was matched for type of surgery with 40 consecutive patients undergoing surgical lung resections during the study period, but without functional respiratory impairment at the PFT (both FEV1 and DLCO above 80% of predicted). Demographic, clinical, surgical, and functional features of both groups, as well as comparison results, are shown in Table 1. Mean age was 65.2 years old, 52 patients (65%) were males. Arterial hypertension and diabetes were the most frequent comorbidities. A total of 40 sub-lobar resections (50%), 36 lobectomies (45%), 2 bi-lobectomies (2.5%), and 2 pneumonectomies (2.5%) were performed. Thoracoscopic approach was adopted in 65 cases (81%). Pulmonary nodules were diagnosed as primary lung cancer in 50 cases (62.5%), secondary lung cancer in 10 (12.5%), and non-neoplastic in 20 (25%). Age, weight, body mass index (BMI), smoke history, chronic obstructive pulmonary disease (COPD), surgical approach, FEV1 (absolute and percentage predicted), Tiffenau index, DLCO (absolute and percentage predicted), and length of hospital stay resulted significantly different between the two groups. There were significantly younger age, lower weight and BMI, higher percentage of active smokers, higher incidence of COPD, lower number of thoracoscopic procedures and longer hospital stay in the group of patients assessed by CPET. Furthermore, the same patients presented lower FEV1, Tiffenau and DLCO values than patients not assessed by CPET, as expected. In the CPET-group, within 30 days from surgery, 4 patients (10%) developed major cardiopulmonary complications, 1 of whom (2.5%) died due to heart failure. Focus on their demographic, functional, and surgical characteristics is reported in Table 2. The control patients experienced 5% major cardiopulmonary complications (2/40) and 0% death rates. In total, 13 major cardiopulmonary complications occurred in 6 patients (0.75%) in the two groups: pneumonia (4 cases), sepsis (1 case), bronchopulmonary infection (2 cases), ARDS (3 cases), arrythmia (2 cases), heart failure (1 case). Both morbidity and mortality rates, although differing between the two groups (10% vs. 5%, and 2.5% vs. 0%, respectively), did not reach statistical significance.

**Table 1:** Patients’ characteristics and comparison between patients with functional pulmonary impairment assessed by CPET (CPET group) and patients without functional pulmonary impairment (non-CPET group). Statistically significant variables are in bold.

Variable	CPET group (*N* = 40)	Non-CPET group (*N* = 40)	*p* value
**Demographics**			
Age, mean (SD)	62.7 (10.6)	67.8 (9.6)	0.02
Gender, male (%)	24 (60)	28 (70)	0.11
Height, mean (SD)	165.7 (7.4)	166.0 (11.4)	0.88
Weight, mean (SD)	63.1 (13.8)	76.8 (16.9)	<0.01
BMI, mean (SD)	22.9 (3.9)	27.6 (4.2)	<0.01
Smoke, never/yes/former (%)	7 (17.5)/22 (55)/11 (27.5)	13 (32.5)/2 (5)/20 (50)	<0.01
**Comorbidities**			
Past lung surgery, yes (%)	7 (17.5)	2 (5)	0.07
COPD, yes (%)	12 (30)	1 (2.5)	<0.01
Asthma, yes (%)	1 (2,5)	5 (12.5)	0.09
ILD, yes (%)	3 (7.5)	0 (0)	0.07
Ischemic cardiopathy, yes (%)	2 (5)	5 (12.5)	0.23
Arrythmia, AF/others (%)	1 (2.5)/4 (10)	0 (0)/1 (2.5)	0.22
Heart failure, yes (%)	0 (0)	1 (2.5)	0.31
Arterial hypertension, yes (%)	17 (42.5)	22 (55)	0.26
Diabetes, yes (%)	5 (12.5)	6 (0.15)	0.74
**Surgical**			
Surgical approach, thoracoscopy (%)	29 (72.5)	36 (90)	0.04
Conversion, yes (%)	4 (10)	3 (7.5)	0.69
Lung resection, sublobar/lobar/bi-lobar/pneumonectomy (%)	20 (50)/17 (42.5)/1 (2.5)/2 (5)	20 (50)/19 (47.5)/1 (2.5)/0 (0)	0.62
Lung cancer histology, primary/secondary/other (%)	24 (60)/4 (10)/12 (30)	26 (65)/6 (15)/8 (20)	0.50
**Functional**			
FEV1, mean (SD)	2.28 (0.72)	2.82 (0.74)	<0.01
FEV1%predicted, mean (SD)	83.60 (21.15)	103.68 (14.26)	<0.01
FVC, mean (SD)	3.38 (0.93)	3.63 (0.91)	0.23
FVC %predicted, mean (SD)	97.88 (21.64)	102.35 (10.11)	0.24
Tiffenau, mean (SD)	85.74 (13.86)	98.12 (16.77)	<0.01
DLCO, mean (SD)	14.65 (3.72)	20.23 (3.99)	<0.01
DLCO %predicted, mean (SD)	60.03 (10.92)	87.65 (8.83)	<0.01
**Outcomes**			
Major cardiopulmonary complications, yes (%)	4 (10)	2 (5)	0.39
Death, yes (%)	1 (2.5)	0 (0)	0.32
Length of hospital stay, mean (SD)	9.00 (8.44)	6.20 (2.24)	0.04

CPET, cardiopulmonary exercise testing; SD, standard deviation; BMI, body mass index; COPD, chronic obstructive pulmonary disease; ILD, interstitial lung disease; AF, atrial fibrillation; FEV1, forced expiratory volume in 1 s; FVC, forced vital capacity; DLCO, diffusing lung capacity for carbon monoxide.

**Table 2:** Characteristics of complicated patients within CPET group.

Variable	Patient 1	Patient 2	Patient 3	Patient 4
Age, years	74	75	60	76
Gender	M	M	M	M
Height, cm	160	167	160	169
Weight, kg	50	75	59	67
BMI, kg/m^2^	19.6	26.9	23.1	23.5
Smoke	Active (60 PY)	Active (50 PY)	Active (50 PY)	Never Smoker
Comorbidities	Arterial hypertension	Arrythmia, arterial hypertension	None	Arterial hypertension, peripheral artery disease
Surgical approach	VATS	Thoracotomy	VATS	Thoracotomy
Lung resection	Sub-lobar	Lobar	Lobar	Bi-lobar
Lung cancer histology	Adenocarcinoma	Squamous	Adenocarcinoma	Squamous
FEV1%predicted	96	98	149	90
FVC %predicted	119	155	155	118
Tiffenau	80	64	94	78
DLCO %predicted	64	44	71	59
Major cardiopulmonary complications	Pneumonia, sepsis, ARDS	Bronchopulmonary infection	Pneumonia, ARDS	Bronchopulmonary infection, pneumonia, ARDS, arrythmia, heart failure
Death	N	N	N	Y
Length of hospital stay, days	39	14	37	29
VO_2_peak, ml/kg/min	22.6	20.2	22.1	20.7
VO_2_peak %predicted	84	92	71	87
AT %predicted	45	46	50	55
VO_2_/W slope, ml/min/watt	10.9	10.2	10.4	9.7
VE/VCO_2_ slope	32.4	40.5	30.4	35.6
P_ET_CO_2_peak, mmHg	37	23	34	32
Alveolar-arterial oxygen gradient	28	58	20	35
HRpeak %predicted	86	102	61	89
O_2_pulse %predicted	99	90	118	98

CPET, cardiopulmonary exercise testing; BMI, body mass index; PY, pack year; VATS, video-assisted thoracoscopic surgery; FEV1, forced expiratory volume in 1 s; FVC, forced vital capacity; DLCO, diffusing lung capacity for carbon monoxide; ARDS, acute respiratory distress syndrome; N, no; Y, yes; VO_2_peak, peak oxygen consumption; AT, anaerobic threshold; VO_2_/W, carbon dioxide production to minute work; VE/VCO_2_, minute ventilation to carbon dioxide production; HR, heart rate; PETCO2, end-tidal carbon dioxide pressure.

Main variables provided by CPET in the cohort population are shown in Table 3. In particular, mean VO_2_peak was 23.14 ± 2.59 ml/kg/min, mean VE/VCO_2_ slope 30.47 ± 4.67. 21 patients (56%) displayed a VE/VCO_2_ slope >30, including 9 over 12 patients affected by chronic obstructive pulmonary disease (COPD). A ventilatory limitation to exercise (defined by a breathing reserve below 15%) was observed in 9 patients. 12 patients (30%) presented a reduced exercise capacity.

**Table 3:** Cohort’s CPET values.

Variable	Mean	SD
VO_2_peak, ml/kg/min	23.14	2.59
VO_2_peak %predicted, %	91.80	14.71
AT, ml/kg/min	13.56	2.88
AT %predicted, %	52.87	12.23
VO_2_/W slope, ml/min/W	11.08	0.97
Work %predicted, %	81.13	12.94
VE/VCO_2_ slope, ratio	30.47	4.67
Breathing reserve, %	23.68	20.54
Alveolar-arterial oxygen gradient, mmHg	30.17	12.34
HRpeak %predicted, %	90.32	12.50
HRpeak, bpm	141.63	19.90
O_2_pulse %predicted, %	98.54	22.45

CPET, cardiopulmonary exercise testing; SD, standard deviation; VO_2_peak, peak oxygen consumption; AT, anaerobic threshold; VO_2_/W, carbon dioxide production to minute work; VE/VCO_2_, minute ventilation to carbon dioxide production; HR, heart rate.

## Discussion

This study shows that respiratory impaired low-risk patients, as stratified by CPET assessment, have postoperative morbidity and mortality rates of 10% and 2.5%, respectively, when undergoing oncological lung resections. Furthermore, it first demonstrates that, when comparing these patients with those matched for surgical procedure without any respiratory functional impairment, no differences exist in terms of mortality and morbidity. However, differences in age, weight, BMI, smoke history, COPD, surgical approach, and length of hospital suggest that they really represent different risk categories.

PFT and CPET are two pillars of the preoperative assessment: FEV1 and DLCO, derived from PFT, allow to split standard-risk patients from those with possible increased risk, whereas VO_2_peak, derived from CPET, further stratify those with possible increased risk in low, intermediate, and high risk. While lower limit of 10 ml/kg/min is a well-established cut-off for extreme perioperative risk, the higher limit allowing for safe surgery has been arbitrary defined ranging from 15 to 20 ml/kg/min (11), historically based on limited past experiences of none or minimum complications after those levels (12–14). The 20 ml/kg/min cut-off later resulted as a safe threshold for major lung resection, leading to morbidity rate lower than 10% and mortality rate of 0% (15–17). Recently, Gooseman and colleagues (18), while assessing outcomes of moderate-risk group undergoing lung surgery, have collaterally reported 19%–30% cardiopulmonary morbidity rate and 0%–4.2% mortality rate, depending on extent of resection, in low-risk patients. Furthermore, Begum and colleagues (19), while evaluating the reliability of CPET in patients undergoing thoracoscopic compared to thoracotomic lobectomy, confirmed these results, detecting cardiopulmonary morbidity rate of 29% and mortality rate of 3.5% in low-risk patients. On the other hand, Kristenson and colleagues (20) have reported 9% rate of cardiopulmonary complications or death in low-risk patients, while investigating early outcomes of moderate-risk patients undergoing lobectomy to evaluate VE/VCO_2_ slope application in improving risk stratification in that category of risk. According to our experience, we reported 10% cardiopulmonary morbidity rate, which is overlapping to that shown by Kristenson and colleagues, although being lower than those presented by the other authors. Indeed, Gooseman and colleagues applied different criteria for defining low-risk patients, adopting ACCP guidelines, thus possibly including patients with greater functional impairment than ours; moreover, among their enrolled patients, more than 70% underwent surgery through open approach, thus potentially explaining the higher rate of complications registered compared to our study, where thoracoscopic approach was applied in more than 70% of cases. On the other hand, higher morbi-mortality rates detected by Begum and colleagues could be explained, besides the enrollment of 80% thoracotomic procedures, by their choice of applying 15 ml/kg/min as threshold for low-risk patients, thus including patients traditionally at moderate risk, who could have increased the incidence of complications. This consideration is supported by the results from Chouinard and colleagues (21), who have shown 10.4% of complication rate in patients undergoing thoracoscopic lobectomy with VO_2_peak >20 ml/kg/min, which is similar to our results. Notably, the percentage of low-risk patients over the total assessed by CPET from our study (23%) is grossly comparable to that reported in literature (18, 19, 21).

According to ESTS/ERS guidelines (3), patients with both FEV1 and DLCO >80% predicted do not have to be further investigated by CPET and can undergo any extent of surgical resection with low risk for perioperative cardiopulmonary complications and deaths, whose incidence rates are about 13% and 0%, respectively (15). Nevertheless, recently, Cundrle and colleagues (22) have selectively analyzed surgical outcomes of this group of patients assessed by CPET, reporting 9% cardiopulmonary morbidity rate, mainly affecting those with lower end-tidal carbon dioxide pressure (P_ET_CO_2_) and increased VE/VCO_2_ slope, and suggesting to perform routinary CPET to identify these patients. Our matched control patients have experienced 5% cardiopulmonary morbidity rate and 0% mortality rate, slightly better but grossly comparable to previous results. We assumed that these rates properly define standard low-risk patients, choosing them as controls. Our cohort of CPET-assessed “low-risk” patients have shown younger age, lower weight and BMI, higher incidence of COPD, lower number of thoracoscopic procedures, and longer length of hospital stay, than controls. Although morbidity and mortality rates did not significantly differ between the two groups, we believe that they belong to physiologically different categories, which might express statistically different outcomes when enrolling greater sample sizes.

The general picture derived from the cardiopulmonary exercise testing in our cohort (Table 3) is that of an overall preserved exercise capacity (mean VO_2_peak 90% predicted), associated with preserved indices of oxygen transport/utilization and normal heart rate response, highlighting a good cardiovascular response to exercise with a prevalent cardiocirculatory limitation to exercise. Not surprisingly, the ventilatory response was more frequently altered, with mean VE/VCO_2_ slope of 30.5, suggesting a certain degree of ventilatory inefficiency.

The 4 patients with VO_2_peak >20 ml/min/kg who experienced major postoperative complications (respiratory infections in all cases) were mostly elderly males with a significant smoking history, normal BMI, reduced DLCO and relatively preserved spirometry (Table 2). Looking at the cardiopulmonary exercise testing variables, all three patients who achieved maximal test showed normal exercise capacity, with preserved cardiovascular response to exercise. Interestingly, mild ventilatory inefficiency was observed in all cases, as indicated by the VE/VCO_2_ slope; a finding which is in line with the emphysematous phenotype, as previously described (23). The two patients with the highest VE/VCO_2_ slopes (35.6 and 40.5) had associated impairment of gas exchange, as evidenced by the increased alveolar-arterial gradient for O2. The fourth patient, on the other hand, exhibited reduced overall exercise capacity (VO_2_peak below the normal limit of 85%), with signs of chronotropic limitation, while presenting ventilatory response at the limit of normality. The ventilatory response in complicated patients suggests a certain degree of ventilatory inefficiency, that can be interpreted as either dysregulated ventilatory drive or increased dead space ventilation, as observed in COPD patients (24). Such a pathophysiological condition might be responsible for the maladaptive cardiopulmonary response in the aftermaths of surgery, leading to higher risk of cardiopulmonary complications. This is in line with previous literature, in which the VE/VCO_2_ slope was identified as a predictor of respiratory complications and death after pulmonary resection surgery (25, 26). Despite being the most used parameter in thoracic surgery, VO2peak is not the only variable provided by CPET. Patients with VE/VCO_2_ slope >34–35 have been demonstrated to experience higher incidence of cardiopulmonary complications and deaths (25, 27, 28) after lung surgery, likely related to increased postoperative ventilation-perfusion mismatch (28, 29), even in patients without moderate-to-severe COPD (27, 30). Our findings confirm the role of other CPET parameters (particularly VE/VCO_2_ slope) as a potential additional variable to refine risk-stratification besides VO_2_peak.

Our study has some limitations which are worth to be considered when interpreting the results. First, its retrospective design could have led to a selection bias. We have enrolled consecutive patients both for cohort and for controls, attempting to minimize this bias. Furthermore, we have decided to match patients based exclusively on type of surgery, in order to detect any difference between the two groups, also demographically. Then, the monocentric nature of the study might prevent generalization of findings to other settings. Finally, the small sample size has precluded multivariable analysis, as well as the comparison between complicated and uncomplicated patients, thus preventing us from drawing definitive conclusions in this way.

Nevertheless, we think that our exploratory findings can have clinical and physiological implications, prompting a thorough preoperative evaluation also in patients historically deemed at low risk for better stratifying their real risk. Further prospective studies with larger sample size are required to confirm our results.

## Conclusions

Thorough preoperative functional assessment is mandatory in surgical lung cancer patients to accurately stratify their perioperative risk of complications or death. Patients with functional pulmonary impairment at PFT, as expressed by FEV1 or DLCO below 80% predicted, but with preserved oxygen consumption at CPET, as manifested by VO_2_peak above 20 ml/kg/min, i.e., the “low-risk” category, have cardiopulmonary morbidity and mortality rates comparable to those of patients without functional pulmonary impairment. Nevertheless, they represent a completely distinct group of individuals, who may deserve further investigations to properly identify those at increased risk. CPET variables overall interpretation may add to the VO_2_peak in identifying higher risk patients, even in this subgroup.

## Data Availability

The raw data supporting the conclusions of this article will be made available by the authors, without undue reservation.
